# Erythropoietin Attenuates Insulin Resistance and Renal Inflammation in High-Sucrose-Treated Rats

**DOI:** 10.3390/ijms26178321

**Published:** 2025-08-27

**Authors:** Hiroe Toba, Denan Jin, Miyuki Kobara, Shinji Takai, Tetsuo Nakata

**Affiliations:** 1 Laboratory of Clinical Pharmacology, Division of Pathological Sciences, Kyoto Pharmaceutical University, Kyoto 607-8412, Japan; kobara@mb.kyoto-phu.ac.jp (M.K.); tetsu@koto.kpu-m.ac.jp (T.N.); 2 Department of Pharmacology, Osaka Medical and Pharmaceutical University, Takatsuki-City 569-8686, Japan; denan.jin@ompu.ac.jp (D.J.); shinji.takai@ompu.ac.jp (S.T.)

**Keywords:** erythropoietin, insulin resistance, inflammation, kidney

## Abstract

Erythropoietin (EPO), clinically used as a therapeutic agent for patients with renal anemia, has been reported to exert tissue protective effects independently of hematopoiesis. Insulin resistance is a pathophysiological condition that causes hypertension, diabetes mellitus, and dyslipidemia, leading to vascular and renal injury. The present study investigated whether EPO would improve insulin resistance and vascular and renal injury in chronic sucrose treatment-induced insulin resistant model rats. Sucrose (12%) was given in drinking water for 10 weeks to induce insulin resistance, and EPO (75 U/kg, 3 times/week) was administered subcutaneously for the last 4 weeks. Responses to the oral glucose tolerance test and values of the homeostatic model assessment for insulin resistance indicated that EPO improved insulin resistance in sucrose-treated rats. Though there were no differences in the expression of phospho-Akt (Ser473)/total Akt among all groups, EPO increased that of phospho-STAT3 (Tyr705)/total STAT3 in the liver. Macrophage infiltration into the adventitial area of the aorta and renal overexpression of monocyte chemoattractant protein-1 in the sucrose-treated group were suppressed by EPO treatment, suggesting anti-inflammatory effects of EPO. EPO also decreased collagen I expression in the kidney. A proinflammatory M1-type macrophage marker, tumor necrosis factor-α, was decreased, and anti-inflammatory M2-type macrophage markers, arginase-1 and interleukin-10, were increased by EPO treatment. These results suggest that EPO improved insulin resistance and vascular and renal inflammation in the setting of insulin resistance.

## 1. Introduction

Erythropoietin (EPO) is a hematopoietic factor that is produced mainly by the kidneys [1]. Recombinant human EPO and its analogues are used clinically for patients with renal anemia [2]. EPO exhibits its hematopoietic property by binding to EPO receptors expressed on the membrane of erythroid progenitor cells and stimulates various intracellular signaling pathways, including the phosphatidylinositol-3-kinase (PI3K)/Akt pathway [3]. The EPO receptor is expressed not only in hematopoietic tissues, but also in non-hematopoietic tissues, such as the vasculature, kidneys, liver, and heart. Accumulating evidence indicates that EPO has pleiotropic protective effects against tissue injury independent of its effect on hematopoiesis through these EPO receptors expressed in non-hematopoietic cells [4,5,6]. Previously, we have reported that a low dose of EPO, which did not affect the hematocrit level, attenuated endothelial dysfunction and macrophage infiltration in aortas of diabetic, hypertensive, and subtotal nephrectomized chronic kidney disease rat models [7,8,9,10,11]. These observations raised the possibility that the application of EPO could be expanded from renal anemia to other diseases such as diabetes mellitus, hypertension, and atherosclerotic diseases.

Insulin binds to the insulin receptor in hepatic and skeletal muscle cells, which activates insulin receptor substrates and the PI3K/Akt pathway, colocalizes glucose transporter type 4 (GLUT4) from the cytosol to the cellular membrane, and takes up circulating glucose [12]. In the settings of insulin resistance, this series of intracellular signaling, so-called insulin signaling, is impaired, and more insulin is needed to decrease blood glucose levels, resulting in hyperinsulinemia [13]. Hyperinsulinemia causes activation of the sympathetic nervous system and the renin–angiotensin system, sodium retention, and vascular remodeling, leading to type 2 diabetes mellitus, hypertension, and dyslipidemia, which are risk factors for atherosclerosis [14,15,16,17,18]. It has been recognized that disorders of insulin signaling occur in cardiovascular and renal cells and play roles in the onset and progression of atherosclerotic diseases in the pathological condition of insulin resistance [19].

Some previous studies reported that EPO treatment improved glucose tolerance in high-fat diet-induced obesity and insulin-resistant model animals, suggesting the possibility that EPO is a novel therapeutic agent for insulin resistance [20,21,22]. In the setting of obesity, adipocytes are hypertrophied, and enlarged adipocytes release inflammatory and pathogenic adipocytokines, such as free fatty acids and tumor necrosis factor-α (TNFα), which induce insulin resistance [23]. However, it has been reported that metabolic syndrome develops even with a normal body mass index (BMI), particularly in Asian people [24]. Therefore, studies into the effects of EPO in a non-obese model with insulin resistance are needed. A high-carbohydrate diet induces insulin resistance in rodents [25,26]. Fructose, included in a high-carbohydrate diet, is the major component that induces insulin resistance [27]. Fructose is metabolized to fructose 1-phosphate by fructokinase and is further metabolized to glyceraldehyde, glyceraldehyde 3 phosphate, pyruvate, and then acetyl-CoA in the liver, resulting in de novo lipogenesis, hepatic accumulation of triglycerides, very-low-density lipoprotein (VLDL) formation, and then, insulin resistance [28]. High-carbohydrate diet-fed animals show non-obese insulin resistance with hyperinsulinemia [13,26]. To investigate the effects of EPO on insulin resistance in the absence of obesity, high-sucrose-induced insulin-resistant model rats were used in the present study.

The present study investigated whether EPO improved insulin resistance in the high-sucrose-induced insulin resistance model rats and whether EPO improved vascular and renal injury. We hypothesized that EPO ameliorates insulin resistance and associated vascular/renal inflammation via STAT3 activation.

## 2. Results

### 2.1. EPO Improved Insulin Sensitivity in High-Sucrose-Induced Insulin Resistance Model Rats

The physiological data are summarized in Table 1. There were no significant differences in body weight among the 3 groups. Water intake and urine volume were increased, and food intake was decreased in the sucrose-treated group. EPO slightly decreased the volume of water intake, but had no effects on food intake and urinary volume. The hematocrit was increased by EPO treatment.

**Figure 1:**
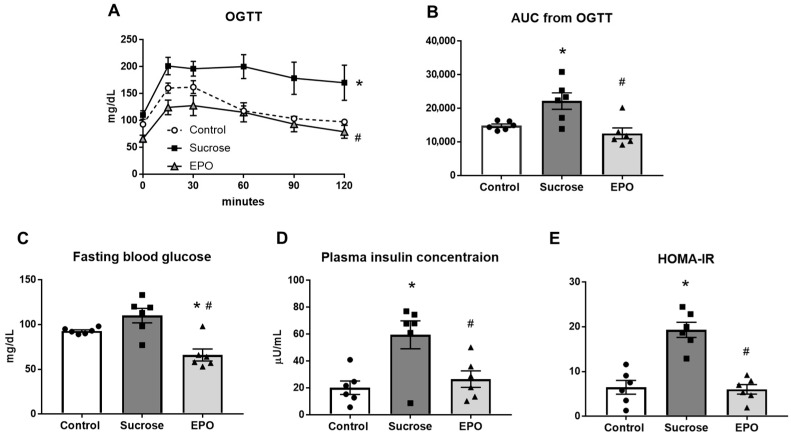
Erythropoietin (EPO) improves insulin resistance in the sucrose-induced insulin resistance model rats. Time-dependent changes in blood glucose levels on the oral glucose tolerance test (OGTT, (**A**)), area under the curve from the OGTT (**B**), fasting blood glucose (**C**), plasma insulin concentration (**D**), and homeostatic model assessment for insulin resistance (HOMA-IR, (**E**)) in the control, sucrose-treated, and EPO-treated groups. After 16 h fasting, fasting blood glucose and plasma insulin concentration were measured, and rats were applied to OGTT. HOMA-IR was calculated using the following formula: fasting blood glucose × plasma insulin concentration/405. Treatment with EPO normalized glucose tolerance, decreased fasting blood glucose, inhibited hyperinsulinemia, and reduced the index of insulin resistance, HOMA-IR. Values are expressed as means ± SEM (n = 6/group). The curves of oral glucose tolerance test of the 3 groups were compared by two-way repeated measures ANOVA, followed by Turkey’s multiple comparisons test. One-way analysis of variance ANOVA followed by Tukey’s multiple comparisons test was used for comparisons among groups. * *p* < 0.05 vs. control, # *p* < 0.05 vs. sucrose.

Chronic sucrose administration significantly blunted the response to oral glucose administration and increased the area under the curve of the oral glucose tolerance test, indicating insulin resistance in the sucrose-treated group. These sucrose-induced changes were suppressed by EPO (Figure 1A,B). Fasting blood glucose was lower in the EPO-treated group than in the control and sucrose-treated groups (Figure 1C). The plasma insulin concentration was increased in the sucrose-treated group, which was reduced by EPO treatment (Figure 1D). In the sucrose-treated group, the values of the homeostatic model assessment for insulin resistance (HOMA-IR), an indicator of insulin resistance, were greater than in the control group. EPO significantly attenuated these changes induced by sucrose, suggesting that EPO improved insulin sensitivity (Figure 1E).

### 2.2. EPO Had No Effects on the Akt Pathway, but Activated the Signal Transducers and Activators of Transcription (STAT)3 Pathway in the Insulin Resistant Rat Liver

**Figure 2:**
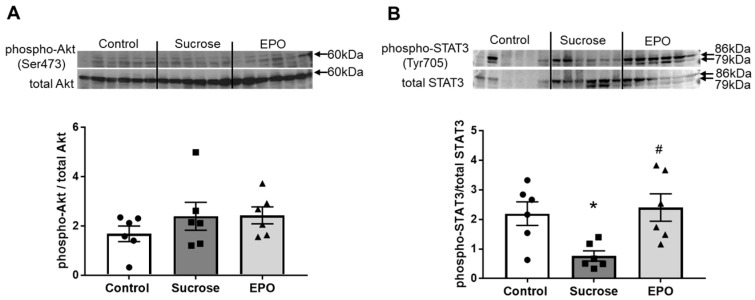
Treatment with erythropoietin (EPO) increases the signal transducer and activator of transcription (STAT)3 pathway in the liver of sucrose-induced insulin resistance model rats. The protein expression of phospho-Akt (Ser473, (**A**)) and phospho-STAT3 (Tyr705, (**B**)) in the livers of the control, sucrose-treated, and EPO-treated groups was assessed by immunoblotting. While there were no significant differences in the expression of phospho-Akt (Ser473) among all groups, treatment with EPO recovered the phosphorylation of STAT3 (Tyr705) in the sucrose-induced insulin resistant rat liver. The expression of each phosphorylated protein was normalized to that of respective total protein. Immunoblot bands were developed by enhanced chemiluminescence and quantified by densitometry. Values are expressed as means ± SEM (n = 6/group). One-way analysis of variance ANOVA followed by Tukey’s multiple comparisons test was used for comparisons among groups. * *p* < 0.05 vs. control, # *p* < 0.05 vs. sucrose.

To investigate the underlying mechanisms of the EPO-induced improvement in insulin sensitivity, intracellular signaling was investigated. There were no differences in the levels of phospho-Akt (Ser473) relative to total Akt in hepatic tissues among the control, sucrose-treated, and EPO-treated groups (Figure 2A). Whereas EPO treatment did not affect the expression of phosphorylated/total Akt, EPO enhanced the levels of phospho-STAT3 (Tyr705) relative to total STAT3 (Figure 2B).

### 2.3. Vascular Inflammation in the Insulin-Resistant Model Rat Was Suppressed in the EPO-Treated Group

**Figure 3:**
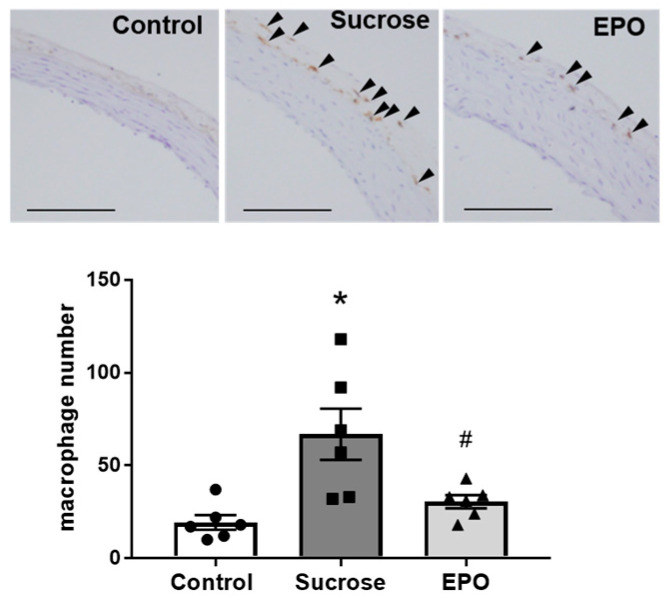
Inflammation in the insulin-resistant rat aorta is suppressed by erythropoietin (EPO) treatment. Macrophages of representative sections of the aorta from rats of the control, sucrose-treated, and EPO-treated groups were detected by immunohistochemical staining. The arrowheads indicate anti-pan-macrophage antibody-positive cells, which were detected using 3,3′-diaminobenzidine chromogen. Bars indicate 50 μm (upper). Number of macrophages that infiltrated into the aortic vessel (lower). Treatment with EPO reduced the number of macrophages infiltrated into adventitial area of the aorta in the sucrose-induced insulin resistant rats. Values are expressed as means ± SEM (n = 6/group). One-way analysis of variance ANOVA followed by Tukey’s multiple comparisons test was used for comparisons among groups. * *p* < 0.05 vs. control, # *p* < 0.05 vs. sucrose.

Macrophage numbers were enhanced in the aortic adventitia from the sucrose-treated group compared with the control group. Treatment with EPO significantly reduced macrophage infiltration in sucrose-treated rats, indicating the anti-inflammatory property of EPO (Figure 3).

### 2.4. EPO Treatment Attenuated Renal Inflammation and Reduced Collagen Content in Insulin-Resistant Model Rats

**Figure 4:**
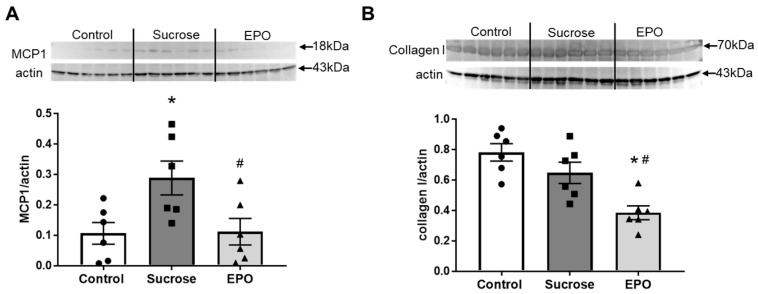
Erythropoietin (EPO) attenuates renal inflammation and reduces collagen contents in sucrose-induced insulin resistance model rats. The protein levels of monocyte chemoattractant protein-1 (MCP1, (**A**)) and collagen I (**B**) in the control, sucrose-treated, and EPO-treated groups were assessed by immunoblotting. EPO reduced the protein expression of MCP1 and collagen I in the sucrose-induced insulin resistant rat kidney. The expression of each protein was normalized to actin. Immunoblot bands were developed by enhanced chemiluminescence and quantified by densitometry. Values are expressed as means ± SEM (n = 6/group). One-way analysis of variance ANOVA followed by Tukey’s multiple comparisons test was used for comparisons among groups. * *p* < 0.05 vs. control, # *p* < 0.05 vs. sucrose.

Chronic sucrose treatment increased monocyte chemoattractant protein-1 (MCP1) expression in the kidney, suggesting that renal inflammation occurred in the insulin-resistant model rats. The increment in MCP1 expression was suppressed by EPO treatment (Figure 4A).

Collagen I is the major component of fibrillar collagens in the kidney. The expression of collagen I in the kidney was lower in the EPO-treated group than in the control and sucrose-treated groups (Figure 4B).

### 2.5. EPO Promoted Macrophage M1 < M2 Polarization in the Kidney of Sucrose-Treated Insulin-Resistant Model Rats

**Figure 5:**
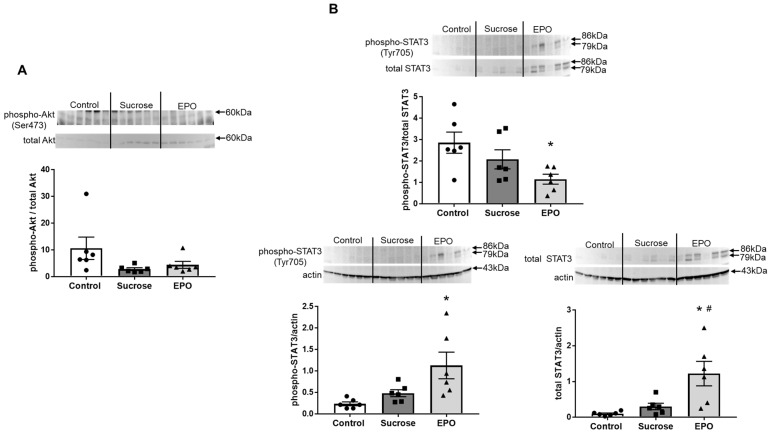
Treatment with erythropoietin (EPO) tends to increase the signal transducer and activator of transcription (STAT)3 pathway in the kidney of sucrose-induced insulin resistance model rats. The protein expression of phospho-Akt (Ser473, (**A**)) and phospho-STAT3 (Tyr705, (**B**)) in the kidneys of the control, sucrose-treated, and EPO-treated groups was assessed by immunoblotting. *A*: The expression of phosphorylated Akt was normalized to that of total Akt. *B*: The expression of phosphorylated STAT3 was normalized to that of total STAT3 (upper) and actin (lower, left). Total STAT3 was normalized by actin (lower, right). While there were no significant differences in the expression of phospho-Akt (Ser473) among all groups (**A**), treatment with EPO reduced the phosphorylation ratio of STAT3 (Tyr705)/total STAT3 in the sucrose-induced insulin resistant rat kidney ((**B**), upper). On the other hand, the protein levels of phospho-STAT3 (Tyr705) and total STAT3, normalized with actin, were increased in the EPO-treated rat kidney. Immunoblot bands were developed by enhanced chemiluminescence and quantified by densitometry. Values are expressed as means ± SEM (n = 6/group). One-way analysis of variance ANOVA followed by Tukey’s multiple comparisons test was used for comparisons among groups. * *p* < 0.05 vs. control, # *p* < 0.05 vs. sucrose.

The expression of phospho-Akt (Ser473) relative to total Akt tended to be lower in the sucrose-treated rat kidney than in the control rat kidney, and EPO had no effects on the expression of phospho-Akt/total Akt (Figure 5A). Whereas the expression of phospho-STAT3 (Tyr705) relative to total STAT3 decreased in the EPO-treated group, the levels of phosphorylated and total STAT3 relative to housekeeping protein actin were upregulated in the EPO-treated rat kidney (Figure 5B).

**Figure 6:**
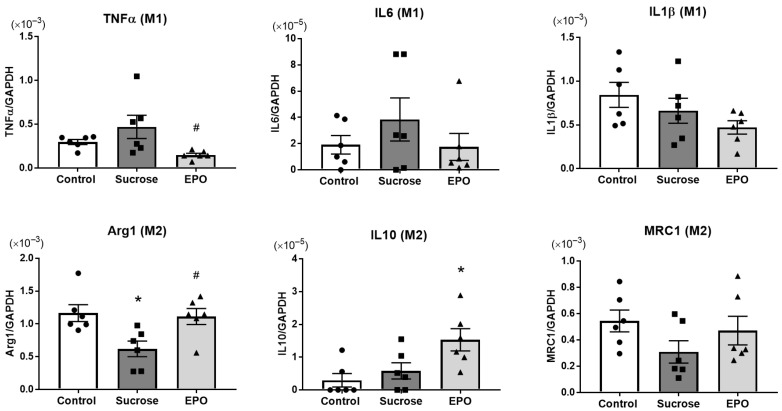
Erythropoietin (EPO) promotes macrophage M1 < M2 polarization in the kidney of sucrose-induced insulin resistance model rats. The mRNA levels of macrophage M1 markers (*A*, tumor necrosis factor-α [TNFα], interleukin-6 [IL6], and interleukin-1β [IL1β]) and M2 markers (*B*, arginase-1 [Arg1], interleukin-10 [IL10], and mannose receptor c-type 1 [MRC1]) in the control, sucrose-treated, and EPO-treated groups assessed by RT-PCR. While there were no significant differences in the mRNA levels of IL6, IL1β, and MRC1, treatment with EPO decreased the mRNA levels of TNFα and increased those of Arg1 and IL10 in the sucrose-induced insulin resistant rat kidney. The expression of each mRNA was normalized to glyceraldehyde-3-phosphate dehydrogenase (GAPDH) and is shown as 2^−∆Ct^. Values are expressed as means ± SEM (n = 6/group). One-way analysis of variance ANOVA followed by Tukey’s multiple comparisons test was used for comparisons among groups. * *p* < 0.05 vs. control, # *p* < 0.05 vs. sucrose.

Because EPO was observed to suppress the overexpression of renal MCP1, the mechanisms of the anti-inflammatory effects of EPO were subsequently examined by investigating the mRNA levels of M1 and M2 macrophage markers by RT-PCR. There were no significant differences in the M1 macrophage markers, such as TNFα, interleukin-6 (IL6), and interleukin-1β (IL1β), between the control and sucrose-treated groups, whereas TNFα and IL6 tended to increase with sucrose treatment. EPO significantly reduced TNFα mRNA levels. The sucrose-treated group showed lower expression of the M2 macrophage marker arginase-1 (Arg1) compared with the control group, and EPO recovered expression of Arg1 to the control level. Although there were no significant differences, the mRNA levels of mannose receptor c-type 1 (MRC1), a marker of M2 macrophages, showed similar changes to those of Arg1. Another M2 macrophage marker, interleukin-10 (IL10), was increased by EPO treatment (Figure 6).

## 3. Discussion

The prevalence of insulin resistance is estimated to range from 15.5% to 46.5% in adults worldwide [29]. One of the major risk factors for insulin resistance is aging, which causes a high proportion of visceral fat, oxidative stress, and mitochondrial dysfunction [30]. Abdominal adiposity leads to release of free fatty acids and proinflammatory cytokines, causing the development of hepatic insulin resistance [31]. The population with adult obesity in 2022 has doubled since 1990, reaching 1 in 8 people in the world [32]. To find a novel therapeutic strategy by drug-repositioning, the effects of EPO were examined in sucrose-induced insulin resistance model rats. The salient findings of the present study were as follows: (1) EPO treatment inhibited chronic sucrose-induced impairment of glucose tolerance; (2) STAT3 signaling in the liver was activated by EPO treatment; (3) macrophage infiltration, which was increased in the insulin-resistant rat aorta, was reduced by EPO treatment; (4) renal expressions of MCP1and collagen I were suppressed by EPO; and (5) EPO promoted macrophage M1 < M2 polarization in the insulin-resistant rat kidney, and the increment in the STAT3 pathway might contribute to the renal protective effect of EPO. This study showed for the first time that EPO attenuates insulin resistance and vascular and renal inflammation and that EPO might be a potential therapeutic tool for patients with insulin resistance.

We previously reported that EPO inhibited oxidative stress and inflammation in the vasculature and kidneys in animal models of hypertension, diabetes, and chronic kidney disease beyond its hematopoietic effects, and that activation of the PI3K/Akt pathway would be the pivotal mechanism of the tissue protective effects of EPO [7,8,9,10,11]. Since the PI3K/Akt pathway is the central pathway in insulin signaling [12], we hypothesized that EPO improves insulin sensitivity by activating the PI3K/Akt pathway. EPO did not increase Akt phosphorylation, and, instead, EPO enhanced hepatic expression of phospho-STAT3 (Tyr705), indicating the activation of the STAT3 pathway. Tsuma et al. demonstrated that 4-week intraperitoneal injection of EPO and a long-acting erythropoiesis-stimulating agent darbepoetin alfa ameliorated glucose tolerance and insulin resistance in mice with high-fat diet-induced obesity [22]. They also found that improvement of lipid metabolism and inflammation via EPO receptor-STAT3 activation in the liver played roles in these beneficial effects of EPO and darbepoetin alfa. The present study, taken together with the previous report, suggests that EPO could improve insulin resistance by activating the hepatic STAT3 pathway.

Chronic vascular inflammation and endothelial injury are the initial steps in the onset and progression of atherosclerosis [33,34]. In the present study, EPO prevented the accumulation of macrophages in the adventitial area of the aorta in the insulin-resistant model rats. Recruitment, adhesion on the endothelial cell surface, and infiltration of monocytes occur through endothelial cells from the luminal site into the vascular wall, resulting in monocyte-macrophage differentiation, phagocytosis of oxidized low-density lipoprotein by foam cells, and formation of atherosclerotic plaque [34,35]. In the present study, macrophages were detected only in the outer adventitial area. There was a possibility that macrophages leaking from micronutrient vessels surrounding the aorta were detected. Recently, it has been reported that accumulation of inflammatory cells in the outer area of the arteries, which is called the artery tertiary lymphoid organ, precedes atherosclerotic plaque formation [36]. Therefore, another possibility is that the very early stage of atherosclerosis was observed. Vascular cells, such as endothelial and smooth muscle cells, and macrophages express EPO receptors, and our previous study showed that EPO inhibited endothelial dysfunction and macrophage infiltration by nitric oxide production from endothelial cells via the PI3K/Akt pathway [7,9,10,11,37]. Further investigations with more observations of vascular injury and the mechanisms of anti-inflammatory effects of EPO are needed in a model of insulin resistance.

Renal injury is a major comorbidity of insulin resistance-induced common diseases, such as diabetes mellitus, hypertension, and dyslipidemia [38]. It has been reported that the temporal administration of EPO attenuates the acute injury caused by ischemia/reperfusion of various organs, including the kidney [4]. Our previous study showed that long-term treatment with EPO exerted renoprotective effects in streptozotocin-induced type 1 diabetes mellitus [8]. Anti-apoptotic properties of EPO via PI3K/Akt are common and pivotal mechanisms of renal protection [39,40,41]. In addition, restoring the contents of endothelial nitric oxide synthase, due to the activation of the PI3K/Akt pathway, contributed to the prevention of diabetic nephropathy by EPO [8]. However, EPO had no effects on the expression of the activated form of Akt in the present study and tended to increase the total and activated forms of STAT3. In high-fat diet-induced obese mice, in vivo EPO treatment reduced proinflammatory M1-type macrophages and increased M2-type macrophages in white adipose tissue [20]. In the same study, the authors observed that in vitro EPO treatment induced STAT3 phosphorylation in macrophages sorted from white adipose tissue, which was prevented in EPO-receptor-depleted cells. Macrophages are classified into two major subtypes: proinflammatory (classically activated) M1 and anti-inflammatory (alternatively activated) M2 macrophages [42]. Macrophages are thought to exist along the M1-M2 spectrum and with mixed phenotypes in vivo [23]. In obese human adipose tissue, the density of CD11c-positive M1 macrophages was found to be correlated with markers of insulin resistance [43]. In obese mice, deletion of CD11c-positive M1 macrophages improved insulin sensitivity [44]. Consistently, diet-induced obesity increased the expression of M1 macrophage markers and decreased that of M2 macrophage markers in mouse adipose tissue, whereas the adipose tissue isolated from lean mice expressed many kinds of M2 macrophage markers [45]. It has been suggested that M1 macrophages facilitate insulin resistance, and M2 macrophages suppress adipose tissue inflammation and insulin resistance [46]. Adipose tissue of obese humans and animals has larger quantities of immune cells, such as macrophages, T cells, B cells, neutrophils, and mast cells, compared to non-adipose tissue, and macrophages are the predominant immune cell type [47]. Inflammation in adipose tissue alters the adipokine secretome, which affects remote organs and tissues, resulting in insulin resistance and eventually cardiovascular and renal diseases. Based on these findings, we hypothesized that macrophage M1 < M2 polarization is related to the EPO-induced renoprotective effects in sucrose-induced insulin resistance model rats and found that EPO decreased the M1 marker TNFα and increased the M2 markers Arg1 and IL10 in the kidney. However, the possibility that the expression of these molecules was detected not only from macrophages, but also other cell types composing renal tissue, such as mesangial cells, podocytes, tubular epithelial cells, fibroblasts, and vascular endothelial cells, cannot be excluded. Future studies into macrophage polarization in the kidney induced by EPO are needed.

As described briefly above, the PI3K/Akt pathway has been reported to be an important pathway for the tissue-protective effects of EPO. Stimulation of the EPO receptor causes phosphorylation of Janus tyrosine kinase (JAK) 2, which activates its downstream PI3K and Akt. Akt has multiple targets including anti-apoptotic signals [48]. Our previous study demonstrated that EPO recovered the expression of phospho-Akt and phospho-GSK3, which were decreased in the subtotal nephrectomized rat aorta [10]. In addition, increases in TdT-mediated dUTP Nick End Labeling (TUNEL)-positive apoptotic cells in diabetic rat kidneys were suppressed by EPO treatment, with increases in phosphorylated Akt [8]. The PI3K/Akt pathway is known to increase the expression and activation of endothelial nitric oxide synthase, causing production of nitric oxide, which inhibits processes associated with atherosclerosis and inflammation [49]. We have previously demonstrated that PI3K/Akt activation-induced nitric oxide production plays pivotal roles in EPO-induced vascular and renal production [8,10,11]. Contrary to the previous observations, EPO did not have any effects on the expression of phospho-Akt and total Akt. Though the expression of phospho-Akt was decreased in disease models, sucrose-treatment had no effects on phospho-Akt expression. In addition, apoptotic cells, detected by TUNEL staining, were rarely detected in the sucrose-treated group. The mild injury with unchanged levels of phospho-Akt might be one of the reasons for results that differed from those of previous studies. Supportively, previous studies from other groups reported that, in the sucrose-fed rat heart, there were no significant differences in either phosphorylated or total Akt expression compared with the control and EPO-treated groups. Another possibility is that overexpressed suppressor of cytokine signaling (SOCS), induced by EPO-induced activation of the PI3K/Akt pathway, might have suppressed the changes in the expression of phospho-Akt and total Akt in the present study.

This study has some limitations. First, it is difficult to conclude that the results observed in the EPO-treated group were independent of hematopoiesis because hematocrit was increased by EPO treatment. In our previous studies, 150 or 75 U/kg (3 times/week, s.c.) of EPO were injected in rat models of type 1 diabetes mellitus, hypertension, and chronic kidney disease, and these doses of EPO did not affect hematocrit levels [7,8,9,10,11]. Therefore, 150 U/kg were first administered for 10 weeks, which unfortunately, increased hematocrit levels. The dose was then reduced to 75 U/kg, and the treatment period was shortened for the last 4 weeks. However, hematocrit was still higher in the EPO-treated group than in the other groups. In contrast, it has been reported that EPO-derived Helix B-surface peptide, which is non-erythrogenic, but retains other functions of EPO, improved obesity and insulin resistance without stimulating hematopoiesis, suggesting that improvement of insulin resistance by EPO in the present study would be, at least in part, independent of its hematopoietic effects [21]. Second, further investigations into other EPO-related intracellular signaling pathways and determinations of target cell types of EPO in each tissue are needed. The third limitation is that the present study lacks mechanistic interpretation because of the absence of causal experiments, and future investigations using STAT3 inhibitors or pharmacological controls are needed. Since Alnaeeli et al. reported that EPO/EPO receptor signaling promotes STAT3 activation with anti-inflammatory effects in adipose tissue macrophages, we hypothesized that STAT3 activation is one of the mechanisms of the effects of EPO [20]. However, it is difficult to determine the role of STAT3 and to exclude the possibility that the changes in STAT3 expression might be subsequent to anti-inflammatory responses because the present study only showed the levels of STAT3 expression. Another limitation is that the present study did not investigate cellular levels. For instance, further experiments with immunocytochemistry and fluorescence-activated cell sorting (FACS) will strengthen the present findings by providing more information about the phosphorylation of Akt and STAT3 and macrophage polarization.

## 4. Materials and Methods

### 4.1. Animals

All animal experiments were performed at Kyoto Pharmaceutical University based on the National Research Council’s and National Institute of Health guidelines and were approved by the Institutional Animal Care and Use Committee of Kyoto Pharmaceutical University (CPCO-17-002). Male Wistar/ST rats (200 g, Shimizu Laboratory Supply, Kyoto, Japan) were housed in an environmentally controlled room with a 12 h light/dark cycle, given standard rodent chow and tap water ad libitum. After adaptation to the environment, the rats were randomized and used for the following experiments. The sample size was decided considering the necessity for statistical analysis and the ethics in animal experiments. Potential confounders such as the order of treatments and the location of animal cages were minimized by randomization. The following indications were monitored for humane endpoints: food and water intake difficulties, moribund symptoms, abnormal appearance over a prolonged period with no visible indications of recovery, 20% or more weight loss over several days. Experiments were reported in accordance with the ARRIVE guidelines 2.0 [50].

### 4.2. Insulin Resistance Model and EPO Treatment

The right kidney was removed under anesthesia (0.375 mg/kg medetomidine, 2.0 mg/kg midazolam, and 2.5 mg/kg butorphanol tartrate, i.p.). After a one-week recovery period, 12% sucrose was administered in drinking water for 10 weeks. In the EPO-treated group, recombinant human EPO (Kyowa Kirin, Tokyo, Japan), at a dose of 75 U/kg, was administered subcutaneously three times per week in the last four weeks. Urine samples were collected using metabolic cages. After fasting for 16 h, the blood samples were collected, and rats were submitted to an oral glucose tolerance test. Under deep anesthesia (0.375 mg/kg medetomidine, 2.0 mg/kg midazolam, and 2.5 mg/kg butorphanol tartrate, i.p.), the hepatic, aortic, and renal tissues were isolated from each rat for the following experiments.

### 4.3. Functional Studies

Hematocrit was measured using a hematocrit tube (Hirschmann, Eberstadt, Germany) and centrifugation at 10,000× *g* for 10 min. The plasma insulin concentration was determined with the use of commercially available kits (Shibayagi, Gunma, Japan).

### 4.4. Oral Glucose Tolerance Test

A glucose pulse (50% glucose, 2 g/kg body weight) was administered by gavage. Blood samples were collected at 0 (immediately before administration), 15, 30, 60, 90, and 120 min after administration. Glucose levels were measured with a commercially available kit (Sanwakagaku, Nagoya, Japan). Insulin sensitivity was assessed with the incremental area over 60 min of the glucose response curve (area under the curve; AUC).

### 4.5. Immunoblotting

Frozen livers and kidneys were homogenized in ice-cold Tris buffer (50 mmol/L, pH7.6) containing protease inhibitors. The tissues were homogenized using a Tissue Lyser II (Qiagen, Venlo, The Netherlands). The homogenates were centrifuged for 20 min at 12,000 rpm, and the supernatants were collected. Total protein levels were measured by the bicinchoninic acid method (Pierce, Thermo Fisher Scientific, Waltham, MA, USA). Proteins (40 μg) were mixed with sample buffer containing β-mercaptoethanol, boiled for 5 min, separated with sodium dodecyl sulfate-polyacrylamide gel electrophoresis, and then transferred to PVDF membranes. The membranes were blocked with 5% bovine serum albumin in TBS with 0.1% Tween20 for 4 h at room temperature and then incubated overnight with primary antibodies for phospho-Akt (1:250, Ser473, Cell signaling technology, Danvers, MA, USA), total Akt (1:500, Cell Signaling Technology), phospho-STAT3 (1:2000, Tyr705, Cell Signaling Technology), total STAT3 (1:2000, Cell Signaling Technology), MCP1 (1:500, Abcam, Cambridge, UK), collagen I (1:1000, Cedarlane, Burlington, ON, Canada), and pan-actin (1:5000, Merck Millipore, Darmstadt, Germany) and then incubated with appropriate horse-radish peroxidase-conjugated secondary antibodies (anti-mouse IgG antibody, 1:2500, Promega, Madison, WI, USA; anti-rabbit IgG antibody, 1:2500, Cell Signaling Technology) for 1 h at room temperature. The bands were detected by enhanced chemiluminescence (Cytiva, Tokyo, Japan).

### 4.6. Histology

Aortas were embedded in freezing compound (Sakura Finetek, Tokyo, Japan). Cross sections (5 μm) were fixed in 4% formaldehyde, and endogenous peroxide activity was quenched in methanol containing 0.3% hydrogen peroxide. Sections were incubated with primary antibody for rat pan-macrophages (BMA Biomedicals, Augst, Switzerland). Signals were detected by the avidin-biotin complex method using the 3, 3′-diaminobenzidine chromogen with hematoxylin counterstaining. The number of positively stained cells per total adventitial area was determined.

### 4.7. Reverse Transcription-Polymerase Chain Reaction (RT-PCR)

Total RNA was extracted from kidneys using the ISOGEN II reagent (Nippon Gene, Tokyo, Japan), and RNA concentration was measured by the ultraviolet light absorbance at 260 nm. Complementary DNA was synthesized using the PrimeScript RT reagent kit (Takara, Kusatsu, Japan). TB Green Premix Ex Taq II (Takara) and Thermal Cycler Dice Real Time System (Takara) were used to perform quantitative real-time PCR to mRNA expressions of TNFα, IL1β, IL6, Arg1, IL10, and MRC1. After denaturing for 30 s at 95 °C, the following PCR profile was repeated for 45 cycles: denaturation for 5 s at 95 °C, annealing for 30 s at 55 °C, and extension for 30 s at 72 °C. The sequences of PCR primers used were as follows: TNFα sense; 5′-TAGCAAACCACCAAGCGGAG-3′, TNFα antisense; 5′-TGAAATGGCAAACCGGCTGA-3′, IL1β sense; 5′-CAGCTTTCGACAGTGAGGAGA-3′, IL1β antisense; 5′-GTCGAGATGCTGCTGTGAGA-3′, IL6 sense; 5′-TCTCCTCTCCGGACTTGTGAA-3′, IL6 antisense; 5′-CTCTCCGCAAGAGACTTCCA-3′, Arg1 sense; 5′-CGGCTTGCGAGATGTGG-3′, Arg1 antisense; 5′-TAGCCGGGGTGAATACTGG-3′, IL10 sense; 5′-TTGAACCACCCGGCATCTAC-3′, IL10 antisense; 5′-CCAAGGAGTTGCTCCCGTTA-3′, MRC1 sense; 5′-TGATTCCGGTCGCTGTTCAA-3′, MRC1 antisense; 5′-GAACGGAGATGGCGCTTAGA-3′. The relative mRNA levels of each molecule were calculated by normalization of the threshold cycle (Ct) values of the target genes to the housekeeping gene glyceraldehyde-3-phosphate dehydrogenase (GAPDH).

### 4.8. Statistics

Data are expressed as the means ± SEM. One-way analysis of variance ANOVA followed by Tukey’s multiple comparisons test was used for comparisons among groups. The curves of oral glucose tolerance test of the 3 groups were compared by two-way repeated measures ANOVA, followed by Turkey’s multiple comparisons test. A value of *p* < 0.05 was considered significant. GraphPad Prism 7 software was used for statistical analysis.

## 5. Conclusions

In conclusion, EPO improved glucose tolerance and vascular and renal inflammation in the setting of insulin resistance. Whereas some previous studies have shown that EPO and EPO-derived peptides improve insulin resistance, they used high-fat diet-induced obese animals [20,21,22]. Individuals with normal body weight can become insulin-resistant, and non-obese insulin-resistant patients have risks for dysglycemia and cardiovascular diseases [51]. The current study provided novel evidence in a non-obese insulin-resistant model. Future additional studies will strengthen the possibility of drug-repositioning of EPO from renal anemia to a comprehensive therapeutic agent for insulin resistance.

## Figures and Tables

**Table 1:** Physiological characteristics of the rats in the control, sucrose, and EPO groups.

	Control	Sucrose	EPO
Body weight (g)	459 ± 6	453 ± 2	461 ± 7
Food intake (g)	15.5 ± 1.2	4.4 ± 0.4 *	5.8 ± 0.8 *
Water intake (mL)	38 ± 1.2	124 ± 2.1 *	116 ± 2.5 *#
Urinary volume (mL)	21 ± 1.0	89 ± 1.3 *	90 ± 2.3 *
Hematocrit (%)	48 ± 0.8	48 ± 0.6	68 ± 0.3 *#

Values are means ± SEM (n = 6/group). * *p* < 0.05 vs. Control, # *p* < 0.05 vs. Sucrose.

## Data Availability

The data generated and analyzed during this study are available upon reasonable request from the corresponding author.
